# Protein Processing in Plant Mitochondria Compared to Yeast and Mammals

**DOI:** 10.3389/fpls.2022.824080

**Published:** 2022-02-02

**Authors:** Malgorzata Heidorn-Czarna, Agata Maziak, Hanna Janska

**Affiliations:** Department of Cellular Molecular Biology, Faculty of Biotechnology, University of Wrocław, Wrocław, Poland

**Keywords:** protein processing, proteases, mitochondria, N-terminomics, COFRADIC, ChaFRADIC, TAILS, limited proteolysis

## Abstract

Limited proteolysis, called protein processing, is an essential post-translational mechanism that controls protein localization, activity, and in consequence, function. This process is prevalent for mitochondrial proteins, mainly synthesized as precursor proteins with N-terminal sequences (presequences) that act as targeting signals and are removed upon import into the organelle. Mitochondria have a distinct and highly conserved proteolytic system that includes proteases with sole function in presequence processing and proteases, which show diverse mitochondrial functions with limited proteolysis as an additional one. In virtually all mitochondria, the primary processing of N-terminal signals is catalyzed by the well-characterized mitochondrial processing peptidase (MPP). Subsequently, a second proteolytic cleavage occurs, leading to more stabilized residues at the newly formed N-terminus. Lately, mitochondrial proteases, intermediate cleavage peptidase 55 (ICP55) and octapeptidyl protease 1 (OCT1), involved in proteolytic cleavage after MPP and their substrates have been described in the plant, yeast, and mammalian mitochondria. Mitochondrial proteins can also be processed by removing a peptide from their N- or C-terminus as a maturation step during insertion into the membrane or as a regulatory mechanism in maintaining their function. This type of limited proteolysis is characteristic for processing proteases, such as IMP and rhomboid proteases, or the general mitochondrial quality control proteases ATP23, *m*-AAA, *i*-AAA, and OMA1. Identification of processing protease substrates and defining their consensus cleavage motifs is now possible with the help of large-scale quantitative mass spectrometry-based N-terminomics, such as combined fractional diagonal chromatography (COFRADIC), charge-based fractional diagonal chromatography (ChaFRADIC), or terminal amine isotopic labeling of substrates (TAILS). This review summarizes the current knowledge on the characterization of mitochondrial processing peptidases and selected N-terminomics techniques used to uncover protease substrates in the plant, yeast, and mammalian mitochondria.

## Introduction

Mitochondria evolved approximately 1.5 billion years ago as double-membrane organelles originating from the bacterial phylum α-Proteobacteria entering a stable endosymbiosis with the host cell (Gray et al., 1999; Gray, 2012). Over the years of evolution, the mitochondrial genome (mitochondrial DNA, mtDNA) has been drastically reduced by relocating most of the genetic information in the nucleus. The mitochondrial genome of plant *Arabidopsis thaliana* encodes 33 proteins (Unseld et al., 1997; Sloan et al., 2018). In comparison, there are only 13 proteins in humans (Anderson et al., 1981; Capt et al., 2016) and eight proteins in yeast *Saccharomyces cerevisiae* encoded by the mtDNA (Foury et al., 1998; Malina et al., 2018). In consequence, most mitochondrial proteins are synthesized on cytosolic ribosomes and imported post-translationally to the mitochondria. Delivery of mitochondrial precursor proteins to the organelle and sorting them into one of the four mitochondrial subcompartments (outer membrane, OM; intermembrane space, IMS; inner membrane, IM; matrix) is achieved by mitochondrial targeting signals such as cleavable N-terminal presequences, several non-cleavable internal and C-terminal signal sequences or cysteine-rich motifs (Table 1) (Schleiff and Becker, 2011). These targeting signals are recognized by specific receptors of the protein import machinery localized in the outer and inner membranes allowing for the correct protein targeting and sorting in the mitochondria (Ghifari et al., 2018; Pfanner et al., 2019). Most mitochondrial proteins contain N-terminal targeting sequences, which are cleaved upon import into mitochondria, generating functional proteins (Teixeira and Glaser, 2013; Poveda-Huertes et al., 2017; Ghifari et al., 2019).

**TABLE 1:** Targeting sequences of nuclear-encoded mitochondrial proteins.

Destination	Signal
OM	• Non-cleavable N-terminal, internal, C-terminal, or multiple anchors. • Non-cleavable β-signal.
IMS	• Cleavable N-terminal presequence with cleavable hydrophobic sorting signal.
IM	• Cleavable N-terminal presequence with non-cleavable hydrophobic sorting signal. • Non-cleavable multiple hydrophobic signals. • Non-cleavable internal presequence-like signal. • Cysteine-rich motif.
Matrix	• Cleavable N-terminal presequence.

*OM, outer membrane; IMS, intermembrane space; IM, inner membrane. Based on Schleiff and Becker (2011).*

In contrast to complete protein degradation, protein processing, often referred to as limited proteolysis, is an essential biological mechanism controlling protein activity, location, or function. Removing the N-terminal mitochondrial presequences is the most common way of protein processing in the mitochondria. However, the processing of mitochondrial proteins can also be achieved by cleaving a peptide from the N- or C-terminus of a protein as a precise maturation step during insertion of a nuclear or mitochondrially encoded protein into the membrane or as a regulatory mechanism in maintaining mitochondrial function. Recently, the identification in the Arabidopsis mitochondrial ribosome fractions of several proteases involved in protein maturation and degradation has suggested that processing of nascent polypeptides may also occur at mitochondrial ribosomes (Rugen et al., 2019).

Mitochondria possess a distinct and highly conserved proteolytic system that includes proteases with sole function in presequence processing and proteases, which show diverse mitochondrial functions with limited proteolysis as an additional one. Mitochondrial proteases often do not have one role, and many show overlapping substrate specificity (Glynn, 2017). Generally, mitochondrial proteases can be classified based on the utilization of ATP for their activity, which gives two groups of proteases: ATP-dependent and ATP-independent (Janska et al., 2010). Members of processing peptidases are present in both groups (Table 2). In this review, we will focus predominantly on processing peptidases and their substrates in the plant, yeast, and mammalian mitochondria. Additionally, we will highlight mass spectrometry-based N-terminomics techniques elucidating mitochondrial protease cleavage sites and uncovering their proteolytic substrates.

**TABLE 2:** Mitochondrial proteases involved in limited proteolysis and their substrates in yeast, mammalian, and plant mitochondria.

Protease (Class)	Yeast			Human			Plants		
	Subunits	Localization	Substrates	Subunits	Localization	Substrates	Subunits	Localization	Substrates
MPP* (Metalloprotease)	Mas1 Mas2	Matrix	Majority of presequence-containing proteins (Burkhart et al., 2015)	PMPCB PMPCA	Matrix	Majority of presequence-containing proteins (Calvo et al., 2017)	β-MPP α-MPP	Inner membrane (integration into the cytochrome *b-c*1 complex)	Majority of presequence-containing proteins
ICP55* (metalloprotease)	Icp55	Inner membrane-bound from the matrix site	Listed in Vögtle et al. (2009) and Venne et al. (2013)	XPNPEP3	Inner membrane-bound from the matrix site	Putative substrates of human and mouse Icp55 homolog are listed in Calvo et al. (2017)	ICP55	Soluble mitochondrial fraction	Listed in Carrie et al. (2015) and Huang et al. (2015)
OCT1* (metalloprotease)	Oct1	Matrix	Listed in Vögtle et al. (2009) and Vögtle et al. (2011)	MIP	Matrix	Putative substrates of human and mouse MIP are listed in Calvo et al. (2017); Cox4, Prx V, OXA1L (Pulman et al., 2021; Sim et al., 2021)	OCT1	Membrane-bound	Listed in Carrie et al. (2015)
IMP* (serine protease)	Imp1 Imp2 Som1	Inner membrane	Cox2, Cyb2, Mcr1, Gut2, Ptc5, Mgr2, Mcp3 (Nunnari et al., 1993; Bauer et al., 1994; Hahne et al., 1994; Chen et al., 1999; Esser et al., 2004; Ieva et al., 2013; Sinzel et al., 2016) cyt *c*1, Prx1 (Jan et al., 2000; Gomes et al., 2017)	IMMP1L IMMP2L	Inner membrane	Smac/DIABLO, (Burri et al., 2005). cyt *c*1, FAD-dependent glycerol-3-phosphate dehydrogenase (Luo et al., 2006)	IMP1a IMP2	Membrane-bound	Unknown
ATP23* (metalloprotease)	Atp23	Intermembrane space	Atp6 (Osman et al., 2007; Zeng et al., 2007)	ATP23	Intermembrane space	Unknown	ATP23	Soluble mitochondrial fraction	Unknown
Rhomboid protease* (serine protease)	Pcp1 (Rbd1)	Inner membrane	Ccp1, Mgm1 (Esser et al., 2002; Herlan et al., 2003; Schäfer et al., 2010)	PARL	Inner membrane	Pink1, PGAM5, Smac/DIABLO, TTC19 (Jin et al., 2010; Sekine et al., 2012; Saita et al., 2017; Spinazzi et al., 2019)	RBL12	Inner membrane	Unknown
*m-*AAA** (metalloprotease)	Yta10 Yta12	Inner membrane	Ccp1, MrpL32, Oxa1, Ilv2 (Esser et al., 2002; Käser et al., 2003; Nolden et al., 2005; Dasari and Kölling, 2016)	SPG7 AFG3L2	Inner membrane	MrpL32, OPA1, OMA1 (Nolden et al., 2005; Ishihara et al., 2006; Almajan et al., 2012; Consolato et al., 2018)	FTSH3 FTSH10	Inner membrane	AtL32 (Kolodziejczak et al., 2018)
*i*-AAA** (metalloprotease)	Yme1	Inner membrane	Atg32, Ilv2 (Wang et al., 2013; Dasari and Kölling, 2016)	YME1L1	Inner membrane	OPA1 (Ehses et al., 2009; Head et al., 2009; Anand et al., 2014)	FTSH4	Inner membrane	Unknown
OMA1* (Metalloprotease)	Oma1	Inner membrane	Unknown	OMA1	Inner membrane	OPA1, DELE1 (Ehses et al., 2009; Head et al., 2009; Anand et al., 2014; Xiao et al., 2014; Guo et al., 2020)	OMA1	Membrane-bound	Unknown

MPP, mitochondrial processing peptidase; ICP55, intermediate cleavage peptidase of 55 kDa; OCT1, octapeptidyl aminopeptidase 1; IMP, inner membrane peptidase; ATP23, ATP synthase 23; m-AAA, matrix-ATPase associated with a variety of cellular activities; i-AAA, intermembrane space-ATPase associated with a variety of cellular activities; OMA1, Overlapping with the m-AAA protease 1. *ATP-independent protease; **ATP-dependent protease.

## N-Terminal Signals Targeting Proteins Into Mitochondria and Chloroplasts

In plant and algal cells, the evolution of protein targeting mechanisms has been especially challenging due to another type of endosymbiotic organelle, a chloroplast. Most chloroplast proteins, similarly to mitochondria, are nuclear-encoded, synthesized as precursors with N-terminal targeting peptides, and imported to the organelle *via* the chloroplast-specific protein import machinery (Shi and Theg, 2013; Wollman, 2016). Mitochondrial targeting peptides (called presequences) and the chloroplast ones (called transit peptides) show differences in total length and primary structure (Bhushan et al., 2006; Holbrook et al., 2016). Transit peptides are usually longer, with an average of 56 residues in Arabidopsis (Carrie et al., 2015), while mitochondrial presequences have an average length of 43 amino acids in Arabidopsis and 45 in *Oryza sativa* (Huang et al., 2009; Carrie et al., 2015). In contrast, yeast and mammalian presequences are shorter and have a length of 30–37 amino acids (Schneider et al., 1998; Carrie et al., 2015).

Mitochondrial presequences tend to form an amphiphilic α-helix (von Heijne, 1986; Roise et al., 1988), whereas chloroplast transit peptides are somewhat unstructured (Bruce, 2001). However, both presequences and transit peptides show remarkably high similarity at the level of amino acid composition (Bhushan et al., 2006). Interestingly, it has been demonstrated that in cells lacking plastids, specific chloroplast transit peptides deliver proteins into mitochondria (Hurt et al., 1986a,b; Cleary et al., 2002). There is also an increasing number of N-terminal dual targeting peptides, called ambiguous presequences, responsible for distributing dual-targeted proteins between chloroplasts and mitochondria (Pujol et al., 2007; Carrie and Whelan, 2013; Baudisch et al., 2014). N-terminal sequence analyses of dual-targeted proteins from Arabidopsis showed their average length of 59 amino acids, which is significantly longer than typical mitochondrial presequences (Carrie et al., 2015).

**FIGURE 1:**
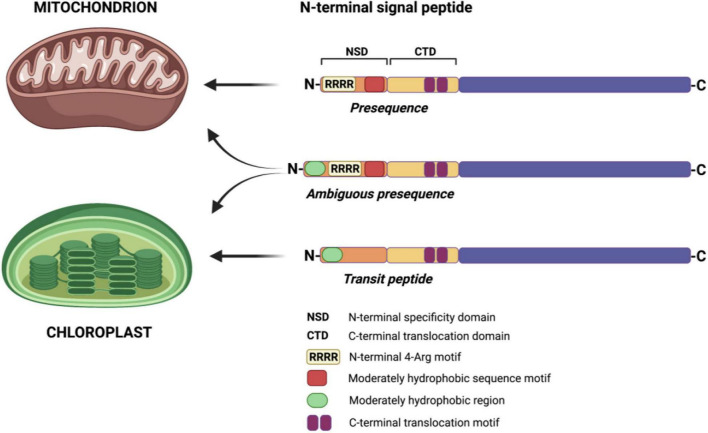
Determinants of a targeting sequence for protein import to mitochondria and chloroplasts in plant cells. N-terminal signal peptides of both mitochondrial and chloroplast proteins contain the N-terminal specificity domain (NSD) and the C-terminal translocation domain (CTD). The NSD determines the specificity of protein targeting, while the CTD of both targeting sequences is interchangeable. In mitochondrial presequences, the N-terminal 4-Arg motif and moderately hydrophobic sequence motif are crucial for protein-specific import into mitochondria. In chloroplast transit peptides, the presence of a moderately hydrophobic region in the NSD is sufficient to target a protein into chloroplasts. In the case of dual-targeted sequences (ambiguous presequences), combining the N-terminal region of transit peptide with mitochondrial presequence results in a protein import to both mitochondria and chloroplasts. Based on Lee et al. (2019) and McKinnon and Theg (2019). Created with BioRender.com.

Studies of mitochondrial and chloroplast signal peptides revealed distinct differences in the first 16 amino acids and that the N-terminal end of presequences is enriched with arginine (Arg, R) residues (Bhushan et al., 2006). The N-terminal arginines have been previously shown to be essential in mitochondrial protein import in plants (Duby et al., 2001). In a recent study, Lee et al. (2019) revealed that within the first 12 amino acids of a presequence, four Arg residues, referred to as the 4-Arg motif, are critical for delivering a protein to mitochondria (Figure 1). Removing the 4-Arg motif changed the targeting specificity from mitochondria to chloroplasts, whereas adding the 4-Arg motif to the N-terminal region of transit peptides blocked targeting a protein to chloroplasts. On the contrary, moderate hydrophobicity at the N-terminal region of transit peptides was sufficient to specify protein import into chloroplasts. Furthermore, the study also revealed that the chimeric targeting peptide containing the N-terminal region of a chloroplast transit peptide and a complete mitochondrial presequence would specify targeting a protein to both organelles (Figure 1) (Lee et al., 2019; Lee and Hwang, 2020). Similar to the earlier findings (Pujol et al., 2007), Lee et al. (2019) have also shown that the presence of conserved Arg residues determines mitochondrial targeting by both mitochondrial and dual-targeting peptides. Further in-depth surveys are still necessary to understand the evolution of dual-targeted sequences leading to a protein targeting into both mitochondria and chloroplasts (Lee and Hwang, 2020).

## Main Mitochondrial Processing Proteases

**FIGURE 2:**
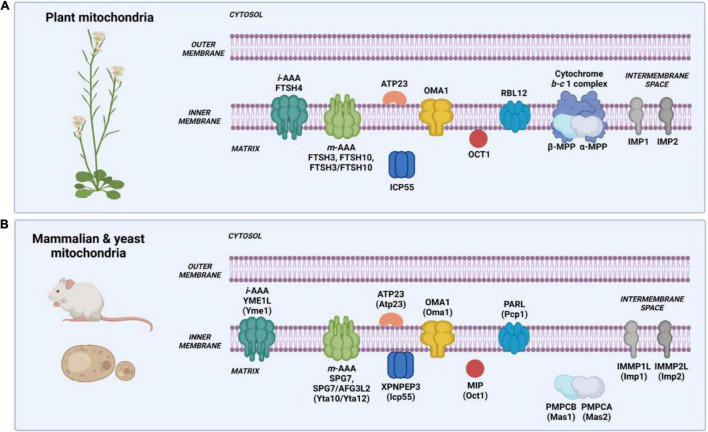
Intramitochondrial localization and nomenclature of proteases involved in protein processing in mammalian, yeast, and plant mitochondria. **(A)** Processing proteases in plant mitochondria. Nomenclature based on the *Arabidopsis thaliana* mitochondrial proteolytic system. **(B)** Processing proteases in mammalian and yeast mitochondria. Nomenclature written in capital letters indicates a mammalian protease, while in brackets – a yeast protease. Created with BioRender.com.

Cleavage of mitochondrial targeting signal is carried out by different processing proteases localized in the mitochondrial matrix and at the inner membrane. The N-terminal presequences of mitochondrial proteins are often removed in the matrix by mitochondrial processing peptidase (MPP) after protein translocation across the two membranes. MPP also cleaves off N-terminal presequences followed by hydrophobic sorting signals in proteins localized in the inner membrane and intermembrane space (Table 1). A hydrophobic sorting signal is removed by additional processing peptidase, the inner membrane protease (IMP). The N-termini of mature proteins typically starts with stabilizing amino acids such as alanine (Ala) and serine (Ser), according to the N-degron pathway (formerly known as the N-end rule pathway) (van Wijk, 2015; Dissmeyer, 2019). However, presequence processing by MPP can generate proteins with destabilizing residues at the newly formed N-terminus. Such destabilized protein intermediates require a second maturation step performed by additional mitochondrial proteases such as intermediate cleavage peptidase 55 (ICP55) or octapeptidyl protease 1 (OCT1), also known as mitochondrial intermediate peptidase (MIP) (Figure 2).

### Mitochondrial Processing Peptidase, MPP

Mitochondrial processing peptidase, MPP, is an ATP-independent metallopeptidase composed of two structurally related subunits generally designated as α-MPP and β-MPP (Mas1 and Mas2 in *S. cerevisiae*, and PMPCB and PMPCA in mammals, respectively), which interact in presequence processing (Table 2) (Sjöling and Glaser, 1998). In Arabidopsis, two genes encode α-MPP, and one encodes β-MPP (Millar et al., 2001). In the MPP complex, the α subunit is responsible for recognizing and binding a presequence. The β subunit, which contains the catalytic site with an inverted Zn-binding motif HxxEH of pitrilysin endopeptidases, performs the actual cleavage (Dvorakova-Hola et al., 2010). Both subunits are essential for the MPP activity (Glaser et al., 1998; Taylor et al., 2001). MPP is a soluble, matrix-localized enzyme in yeast and mammalian mitochondria (Figure 2B) (Hawlitschek et al., 1988; Ou et al., 1989). In plants, the localization of MPP is unique as both α-MPP and β-MPP subunits are integrated into the Cytochrome *b-c*1 complex (complex III) of the respiratory chain as Core proteins facing the matrix (Figure 2A) (Braun et al., 1992; Eriksson and Glaser, 1992; Emmermann et al., 1994; Glaser et al., 1994; Sjöling et al., 1996). In yeast, the complex III accessory subunits Cor1 and Cor2, which show homology to β-MPP and α-MPP, have lost the MPP enzymatic activity; however, in mammals, the complex III preserves basal activity to only one known substrate, the Rieske protein (Taylor et al., 2001; Gakh et al., 2002). Studies using different plant species such as potato (*Solanum tuberosum*), wheat (*Triticum aestivum*), and spinach (*Spinacia oleracea*) showed that despite being an integral component of Cytochrome *b-c*1 complex, the processing activity of MPP is independent of electron transfer (Emmermann et al., 1994; Braun et al., 1995; Eriksson et al., 1996). Recently, using single-particle cryo-electron microscopy (cryo-EM), Maldonado et al. (2021) determined the structure and atomic model of the complex III dimer (CIII_2_) in the mung bean (*Vigna radiata*) mitochondria. For the first time, structural details of the MPP α and β subunits within the plant complex III were demonstrated. The authors showed that each complex III monomer contains an MPP-α/β heterodimer. Within the MPP complex, the α and β subunits form a large central cavity with a highly negative surface, which interacts with positively charged presequences. The cryo-EM map also shows specific density corresponding to Zn^2+^ ion at the catalytic site of the β subunit. Furthermore, the atomic model of plant CIII_2_ revealed that extended N-termini of MPP subunits span across the complex dimer and provide additional contact to its membrane subunits (Braun, 2021; Maldonado et al., 2021). These data strongly confirm the uniqueness of complex III in plants, functioning as a respiratory enzyme and a processing peptidase.

Approximately 80% of presequences have Arg residue at the −2 (−2R) or −3 (−3R) position relative to the mature protein N-termini, which is surrounded by a loosely conserved motif of R-X↓X or R-X-F/Y/L↓A/S-X, respectively (Schneider et al., 1998; Zhang et al., 2001). The third additional group lacks a conserved Arg (no-R) close to the maturation site; however, in plants *A. thaliana* and *O. sativa*, there is a consensus sequence of F/Y↓S/A for no-R group of proteins (Huang et al., 2009). The fourth group contains Arg in position −10 (−10R) with a consensus motif of R-X↓F/L/I-X_2_-T/S/G-X_4_↓X (Isaya et al., 1991). The −2R, −3R, and no-R motifs were found in presequences of all studied organisms; however, the −10R motif is absent in plants (Zhang et al., 2001; Huang et al., 2009). Studies have shown that the −3R motif is the most common cleavage motif in plants, whereas, in yeast and mammals, the most common are −2R and −3R motifs (Huang et al., 2009; Calvo et al., 2017). The ambiguity of the presence of −2R and −3R consensus motifs was later clarified by understanding that the −3R motif results from a subsequent cleavage of amino acid by ICP55 (Vögtle et al., 2009; Carrie et al., 2015; Huang et al., 2015). Furthermore, the cleavage site in the −10R motif was later related to the OCT1 protease (Vögtle et al., 2011).

It has long been assumed that MPP cleaves preproteins at a specific site. Analysis of experimentally identified Arabidopsis presequences with more than 50 amino acids, thus longer than the average length of the *A. thaliana* mitochondrial presequences (43 amino acids), showed that most of them are likely to possess an additional MPP recognition site (Huang et al., 2009). Indeed, the mitochondrial FTSH4 protease precursor is cleaved by MPP at two different sites (Kmiec et al., 2012). The two-step processing carried out by MPP has been previously reported for yeast and human frataxin (Branda et al., 1999; Cavadini et al., 2000) and ATP synthase subunit 9 in *Neurospora crassa* (Schmidt et al., 1984). Using the precursor of mitochondrial tandem protein Arg5,6 as a model substrate from *S. cerevisiae* mitochondria, Friedl et al. (2020) have demonstrated that MPP not only removes the N-terminal targeting sequence from this precursor tandem protein but is also required for specific internal processing generating two functional enzymes, Arg5 and Arg6. Further, *in silico* searching for internal matrix targeting signal-like sequences (iMTS-L) and canonical MPP cleavage sites in mitochondrial precursor proteins with composite structure in different species (Atp25 from *S. cerevisiae* and *Emericella nidulans*, Etp1, Rsm22-Cox11, and the uncharacterized SPAC22A12.08c from *Schizosaccharomyces pombe*, as well as RPS14 from *Oryza sativa japonica*) led to the identification of both types of motifs in these organisms (Friedl et al., 2020). This study indicates that internal precursor processing by MPP is conserved among fungi and plants, and possibly other eukaryotes.

### Intermediate Cleavage Peptidase of 55 kDa

Intermediate cleavage peptidase of 55 kDa (ICP55) is a highly conserved ATP-independent metalloprotease belonging to the aminopeptidase P family (Naamati et al., 2009; Vögtle et al., 2009). Yeast and mammalian ICP55 orthologs are peripherally attached to the mitochondrial inner membrane from the matrix site (Vögtle et al., 2009). In plants, ICP55 has been found in the soluble mitochondrial protein fraction (Migdal et al., 2017) (Table 2 and Figure 2). Interestingly, in yeast, the isoform of Icp55 has also been identified in the nucleus (Naamati et al., 2009), while in humans in the cytosol (Ersahin et al., 2005; Singh et al., 2017).

The stabilizing function of ICP55 was first shown in yeast and human (Vögtle et al., 2009; O’Toole et al., 2010). The protease cleaves a single destabilizing amino acid such as tyrosine (Tyr, Y), leucine (Leu, L), and phenylalanine (Phe, F) at the −2R consensus site (Y/L/F↓S/A) of MPP-generated N-termini, leaving mature proteins with serine (Ser, S), alanine (Ala, A), or threonine (Thr, T) (Vögtle et al., 2009; Teixeira and Glaser, 2013). The analysis of the Arabidopsis mitochondrial N-terminal proteome uncovered that plant ICP55 recognizes consensus cleavage motif of RX(F/Y/I/L)↓(S/A)(S/T), which is remarkably similar to the yeast and mammalian processing sites, and showed that F, Y, and L are the most abundant amino acids removed by the Arabidopsis ICP55 (Carrie et al., 2015; Huang et al., 2015). Notably, in contrast to yeast, the Arabidopsis ortholog is responsible for processing more protein substrates, i.e., approximately 52% of Arabidopsis mitochondrial proteins are processed by ICP55, while only 12% in *S. cerevisiae* (Carrie et al., 2015; Calvo et al., 2017). Furthermore, plant ICP55 protease is also responsible for the cleavage of non-R group proteins (Carrie et al., 2015; Huang et al., 2015). In plants, ICP55 might also cleave some protein substrates twice. The identification of three different N-termini of the mitochondrial acyl carrier protein 3 led to the conclusion that this protein is first cleaved by MPP and then twice by ICP55, leaving the stable amino acid at the N-terminus. Yet, it is also possible that the third cleavage is performed by some unknown protease (Carrie et al., 2015).

The deletion of *ICP55* in yeast and plants had different biological consequences: while the *icp55*Δ yeast mutants were defective in growth on non-fermentable medium at elevated temperature (37°C) (Vögtle et al., 2009), the Arabidopsis *icp55* T-DNA insertional lines were indistinguishable from wild-type plants under different growth conditions (long-day and short-day photoperiod, elevated temperature of 30, 37, or 40°C) (Carrie et al., 2015; Huang et al., 2015; Migdal et al., 2017). On the other hand, in both yeast and Arabidopsis, the loss of ICP55 enhanced *in vitro* mitochondrial protein degradation in the mutant compared to the wild-type (Vögtle et al., 2009; Huang et al., 2015). The functional complementation assay showed that the Arabidopsis ICP55 could substitute for the yeast homolog (Migdal et al., 2017).

### Octapeptidyl Aminopeptidase 1

Octapeptidyl aminopeptidase 1 (OCT1 in Arabidopsis, Oct1 in *S. cerevisiae*, MIP (mitochondrial intermediate peptidase in mammals) is, like MPP and ICP55, an ATP-independent metalloprotease, which was first characterized in rat liver mitochondria and initially named as matrix processing protease II to distinguish it from MPP (Kalousek et al., 1988). In yeast and mammals, the protease is a soluble protein located in the mitochondrial matrix (Isaya et al., 1992; Kalousek et al., 1992), while in plants, it is a membrane-bound enzyme (Table 2 and Figure 2) (Migdal et al., 2017). Oct1 cleaves eight amino acids (octapeptides) from the MPP-generated N-terminal sequences in the protein substrates, which contain Arg in the −10 position from the MPP recognition site (Isaya et al., 1992; Vögtle et al., 2011). Interestingly, the presence of Arg in position −10 of Oct1 substrates is not always a strict requirement as some precursor proteins contain lysine (Prx1, Peroxiredoxin 1) or cysteine, alanine, or aspartate (Imo32, Intermediate cleaved by mitochondrial octapeptidyl aminopeptidase 1) at this position (Vögtle et al., 2011). In plants, mitochondrial presequences lack the −10R motif (Huang et al., 2009), and the identified small set of OCT1 substrates in the Arabidopsis mitochondria did not show the presence of that motif (Carrie et al., 2015). Interestingly, Carrie et al. (2015) also indicated that Arabidopsis OCT1, aside from its processing activity following MPP, can also process mitochondrial presequences without the prior cleavage by MPP. The *in vitro* import into Arabidopsis mitochondria has shown that the B13 subunit of complex I is cleaved exclusively by OCT1 after its insertion into complex I (Carrie et al., 2015). The Arabidopsis plants lacking OCT1 protease did not show any phenotypic and mitochondrial respiratory activity alterations (Carrie et al., 2015; Migdal et al., 2017), in contrast to the *oct1*Δ yeast mutant, which was respiratory deficient when grown on a non-fermentable carbon source (Isaya et al., 1994). The functional complementation experiment showed that the plant homolog of Oct1 could not restore respiratory function in the *oct1*Δ yeast mutant suggesting no conservation between the yeast and plant Oct1 proteases (Migdal et al., 2017).

### Inner Membrane Protease

Mitochondrial precursor proteins destined to the IMS or facing the IMS often contain the hydrophobic sorting signal, which is cleaved off by the mitochondrial inner membrane protease (IMP). IMP is an integral protein embedded in the inner membrane, with its catalytic C-terminus facing the IMS (Figure 2) (Nunnari et al., 1993). The enzyme can process both nuclear and mitochondrial encoded proteins, and the cleavage is usually preceded by the processing of a substrate protein by MPP (Mossmann et al., 2012). Recent studies by Gomes et al. (2017) demonstrated that in yeast, proteolytic maturation of Prx1 involves three proteases, MPP, Oct1, and Imp, which control its submitochondrial localization.

Inner membrane protease has been so far primarily studied in yeast *S. cerevisiae*, in which it exists as a heterodimeric complex consisting of two catalytic subunits, Imp1 and Imp2, and the auxiliary protein Som1, required for the catalytic activity of Imp1 (Schneider et al., 1991; Nunnari et al., 1993; Esser et al., 1996). Imp1 and Imp2 belong to the same serine protease family, yet they recognize a distinct set of substrates (Table 2) (Nunnari et al., 1993). The repertoire of known protein substrates of Imp1 and Imp2 is relatively small. Only seven protein substrates of Imp1 have been identified in yeast mitochondria, with Mcp3 protein lately reported as the first known mitochondrial outer membrane protein processed by Imp1 protease (Table 2) (Sinzel et al., 2016). Interestingly, Imp1 not only processes N-terminal sorting signals but can also cleave off the C-termini. For example, the enzyme removes the C-terminal targeting sequence of the TIM23 subunit Mgr2 and promotes proper assembly of the complex (Ieva et al., 2013).

For the moment, there are only two known Imp2 substrates, cytochrome *c*1 (cyt *c*1, subunit of complex III) and Prx1 (Table 2). Sorting of Prx1 into the IMS requires the cleavage of the protein by Imp2 alone. Alternatively, the localization of Prx1 into the mitochondrial matrix requires the cleavage by MPP followed by the processing by Oct1 protease (Gomes et al., 2017).

Mammalian IMP proteases (in human IMMP1L and IMMP2L, Table 2) have been studied mainly by expression in yeast. So far, there are only three known IMP substrates identified *in vitro*, such as Smac/DIABLO cleaved by the mouse IMP1 (Burri et al., 2005), as well as cyt *c*1 and an ortholog of yeast Gut2, FAD-dependent glycerol-3-phosphate dehydrogenase cleaved by IMP2 in the mouse mitochondria (Table 2) (Luo et al., 2006). Cyt *c*1 is a proteolytic substrate for the yeast Imp2 and mammalian IMP2; on the other hand, glycerol-3-phosphate dehydrogenase has switched from Imp1 in yeast to IMP2 in mammals.

Comparative sequence analyses of the yeast and plant proteolytic system components revealed that the Arabidopsis genome contains six putative Imp homologs (Kwasniak et al., 2012), and among them, two have been analyzed (IMP1a, At1g53530; IMP2, At2g31140) (Migdal et al., 2017). Both IMP1a and IMP2 showed mitochondrial localization, and IMP1a has been found in the membrane fraction, similarly to the yeast counterpart (Figure 2). The morphology, development, and mitochondrial respiration of the Arabidopsis *imp1a* T-DNA insertional line was comparable to the wild-type plants (Migdal et al., 2017). On the contrary, yeast *imp1*Δ and *imp2*Δ knockout mutants showed respiratory growth defects on the non-fermentable carbon sources (Burri et al., 2005). However, the Arabidopsis IMP1a and IMP2 orthologs could not restore the respiratory defects in the functional complementation assay, implying a functional divergence of the yeast and plant IMP (Migdal et al., 2017). No physiological plant IMP substrates have been identified to date.

## Mitochondrial Proteases With Additional Function of Limited Proteolysis

### ATP23 Protease

The functional analysis of ATP23 metalloprotease has been primarily performed in yeast. The protease, which localizes to the IMS, shows diverse functions ranging from precursor protein processing and chaperone activity to protein turnover control (Mossmann et al., 2012). In the *S. cerevisiae* mitochondria, Atp23 mediates the proteolytic cleavage of the presequence of Atp6, the mitochondrially encoded subunit of ATP synthase, after its insertion into the inner membrane (Osman et al., 2007; Zeng et al., 2007). Atp23, together with Atp10, also works as a chaperone in the correct assembly of Atp6 into the ATP synthase complex (Osman et al., 2007; Zeng et al., 2007). Furthermore, Atp23 participates in the degradation of Ups1, the IMS protein involved in regulating the distribution of cardiolipin and phosphatidylethanolamine in the mitochondrial inner membrane (Potting et al., 2010) (Table 2).

The function of ATP23 in plant and mammalian mitochondria is still unknown. The mammalian Atp6 ortholog is synthesized without the N-terminal targeting signal characteristic for the yeast Atp6, indicating that in mammals, this protein is not under the control of ATP23 (Zeng et al., 2007). The Arabidopsis ATP23 has been found in the soluble mitochondrial protein fraction, but the experiments in obtaining T-DNA insertional lines in the *ATP23* gene were unsuccessful (Migdal et al., 2017). In yeast, the deletion of the *ATP23* gene results in respiratory deficiency when the cells are grown on a non-fermentable carbon source (Osman et al., 2007; Zeng et al., 2007). However, the complementation of the *atp23*Δ yeast mutants with the Arabidopsis ATP23 protein did not restore yeast respiratory function, and the plant ATP23 could not process yeast Atp6 (Migdal et al., 2017). These findings raise the possibility that ATP23 acts on different substrates in plants and mammals.

### Rhomboid Protease

Rhomboids are integral membrane ATP-independent serine proteases. This class of proteases is fascinating since it is involved in specific proteolysis, known as regulated intramembrane proteolysis (RIP), in a membrane environment. RIP is a mechanism by which a membrane-anchored protein substrate is cleaved at its transmembrane region by a rhomboid protease within the lipid bilayer and further released as an active protein (Wolfe and Kopan, 2004). Members of this protease family have been shown to localize to mitochondria in yeast, plants, and mammals (Table 2) (McQuibban et al., 2003, 2006; van der Bliek and Koehler, 2003; Kmiec-Wisniewska et al., 2008; Adamiec et al., 2017).

In yeast *S. cerevisiae*, the mitochondrial rhomboid protease Pcp1 (Processing of cytochrome *c* peroxidase) is involved in proteolytic cleavage of two substrates, the cytochrome *c* peroxidase (Ccp1) (Esser et al., 2002) and mitochondrial genome maintenance 1 (Mgm1) (Table 2) (Herlan et al., 2003; Schäfer et al., 2010). Besides being cleaved by Pcp1, the proteolytic maturation of Ccp1 and Mgm1 requires the activity of additional proteases. Before cleavage by Pcp1, Ccp1 is integrated into the inner membrane as a precursor protein through the chaperone activity of the *m*-AAA protease (Schäfer et al., 2010). The second substrate, Mgm1, first undergoes processing by MPP, followed by further proteolytic cleavage by Pcp1 (Herlan et al., 2003).

Interestingly, the mammalian ortholog of the yeast Pcp1, PARL (presenilin-associated rhomboid-like) protease, does not mediate processing of the Mgm1 homolog OPA1 (optic atrophy 1) (Duvezin-Caubet et al., 2007; Anand et al., 2014). Both Mgm1 and OPA1 are GTPases involved in the mitochondrial fusion and cristae formation; however, in mammals, the proteolytic processing of OPA1 is controlled by two other inner membrane proteases, the *i*-AAA YME1L and OMA1 (Griparic et al., 2007; Head et al., 2009; Anand et al., 2014). Instead, PARL cleaves two proteins known to play a role in mitophagy, kinase PINK1 (phosphatase and tensin (PTEN)-induced kinase 1) (Jin et al., 2010; Meissner et al., 2011) and phosphatase PGAM5 (phosphoglycerate mutase family member 5) (Sekine et al., 2012). A recent *in vitro* study reported that PARL is also required for the cleavage of TTC19, a subunit of complex III, and the pro-apoptotic protein Smac-DIABLO (Table 2) (Saita et al., 2017). Impaired proteolytic maturation and expression of TTC19 in the mitochondria of *Parl*^–/–^ mouse brain tissue has been recently demonstrated (Spinazzi et al., 2019).

Knowledge concerning the function of mitochondrial rhomboid proteases in plants is still limited. Out of four predicted as mitochondrially targeted rhomboid-like proteases in *A. thaliana* (Adamiec et al., 2017), only one ortholog of the yeast Pcp1, the RBL12 rhomboid protease, has been experimentally characterized in plant mitochondria (Kmiec-Wisniewska et al., 2008). The mitochondrial localization of the RBL6, RBL15, and RBL16 rhomboid homologs needs to be verified (Adamiec et al., 2017). The complementation of the yeast *pcp1*Δ mutants with the Arabidopsis *RBL12* open reading frame indicated that the plant ortholog does not process the yeast substrates, Ccp1 and Mgm1. These results imply that despite the high similarity at the amino acid sequence level between the yeast and Arabidopsis mitochondrial rhomboids, the substrate recognition and processing mechanism by plant RBLs evolved differently (Kmiec-Wisniewska et al., 2008). It is especially interesting since yeast substrates were processed *in vitro* by the mammalian ortholog, PARL protease (McQuibban et al., 2003).

In plant mitochondria, inner membrane carrier proteins possess an N-terminal extension removed in a two-step processing upon import into the organelle (Murcha et al., 2004). It has been demonstrated that the first cleavage is performed by MPP, while the second processing probably occurs in the IMS and is carried out by an undefined yet serine protease. The authors proposed that a rhomboid protease may play a role in the second cleavage (Murcha et al., 2004).

### Mitochondrial Inner Membrane Metallopeptidases: ATPase Associated With Various Cellular Activities Proteases and OMA1

Two highly conserved hexameric AAA (ATPase associated with various cellular activities) proteases, *i*-AAA and *m*-AAA, have been found in the mitochondrial inner membrane of virtually all eukaryotes. The *i*-AAA exposes its catalytic center to the IMS, whereas *m*-AAA is directed toward the mitochondrial matrix. Both AAA proteases contain an AAA domain and the proteolytic domain with a conserved Zn^2+^-binding motif (HExxH) within a single subunit. The AAA domain, which has a chaperone-like activity, binds and hydrolyzes ATP and delivers substrates to the proteolytic center (Glynn, 2017; Opalinska and Janska, 2018).

In yeast, the *m*-AAA proteases are organized as hexameric complexes composed of the Yta12 and Yta10 subunits (Arlt et al., 1996). The mammalian *m*-AAA forms either homo-oligomeric complexes consisting of AFG3L2 subunits only or hetero-oligomeric complexes composed of AFG3L2 and paraplegin (also known as SPG7) (Koppen et al., 2007). Similarly, plant *m*-AAA complexes are composed of FTSH3 and FTSH10 subunits that can form either homo- or hetero-oligomers (Piechota et al., 2010). In contrast, the *i*-AAA protease is built from six subunits of Yme1 in yeast and YME1L in mammals (Weber et al., 1996). There is also only one *i*-AAA protease in plants, FTSH4 (Table 2 and Figure 2) (Gibala et al., 2009; Smakowska et al., 2016). Earlier studies reported the presence of another *i*-AAA complex, FTSH11, in the mitochondria and chloroplasts of plants grown under short-day photoperiod (Urantowka et al., 2005); however, recent proteomic analyses demonstrated that FTSH11 is exclusively localized to the chloroplasts (Adam et al., 2019), at least when plants were grown under long-day conditions for 4 weeks.

The *i*-AAA and *m*-AAA proteases play a major role in the general quality control of mitochondrial proteome (mtPQC, mitochondrial protein quality control). The proteases remove misfolded, mislocalized, non-assembled, or damaged proteins by breaking them into small peptide fragments (Leonhard et al., 2000). This rapid degradation prevents the formation of potentially toxic protein aggregates within the organelle (Maziak et al., 2021). Aside from their critical role in the mtPQC, the *i*-AAA and *m*-AAA proteases also perform highly specific proteolytic reactions by controlling the life span of regulatory proteins involved in key metabolic pathways (Quiros et al., 2015). Generally, the substrate spectrum of *i*-AAA and *m*-AAA is relatively broad as the proteases degrade not only the IM protein substrates but also the OM and IMS (*i*-AAA), as well as the matrix (*m*-AAA) localized proteins (Opalinska and Janska, 2018; Song et al., 2021).

In recent years, several studies have reported the role of *i*-AAA and *m*-AAA in limited proteolysis. One of the best-known examples is the maturation of MrpL32 protein, a nuclear-encoded subunit of the mitochondrial ribosome, which requires cleavage of the N-terminal presequence by the *m*-AAA protease (Table 2). This process is crucial for ribosomal biogenesis and, in consequence, mitochondrial translation and OXPHOS formation. In yeast, the processing of MrpL32 is postulated to be a central function of the *m*-AAA protease as the synthesis of mitochondrially encoded proteins was strongly impaired in the cells lacking *m*-AAA, and the cells were respiratory incompetent (Nolden et al., 2005). Similarly, a decreased rate of mitochondrial protein synthesis and defective ribosome assembly associated with the lack of MrpL32 processing have also been observed in the AFG3L2 or paraplegin-deficient mammalian mitochondria (Nolden et al., 2005; Almajan et al., 2012). Lately, Kolodziejczak et al. (2018) have shown that the Arabidopsis *m*-AAA proteases, FTSH3 and FTSH10, are able to process plant mitochondrial AtL32 protein, a homolog of the yeast and mammalian MrpL32 (Table 2). In addition, *in organello* protein synthesis revealed that the translation of mitochondrially encoded proteins was strongly impaired in the Arabidopsis mutant lacking both FTSH3 and FTSH10 proteases, but not in plants lacking one of the *m*-AAA. Taken together, the specific processing activity of *m*-AAA toward the ribosomal protein is conserved across diverse eukaryotes, including yeast, mammals, and plants. The processing activity of the *m*-AAA protease has also been shown toward another substrate, the mitochondrial protease OMA1. In mammals, the *m*-AAA AFG3L2 subunit is involved in the proteolytic processing of the 60-kDa precursor form of OMA1 to its mature 40-kDa protein (Consolato et al., 2018).

The question is, how does the *m*-AAA protease distinguish partial processing from a complete protein degradation? It has been shown for MrpL32 that the *m*-AAA protease does not recognize a specific cleavage site. Instead, it starts degrading a protein from its N-terminal unstructured end until reaching a tightly folded C-terminal part of MrpL32 harboring a conserved Cys-rich zinc-binding motif (Bonn et al., 2011). The formation of a folded domain terminates the degradation of a protein and induces the release of mature MrpL32. Plant mitochondrial ribosomal protein AtL32, similarly to yeast MrpL32, contains a Cys-rich sequence motif essential for ribosomal protein processing (Kolodziejczak et al., 2018). It is also possible that partial processing of other mitochondrial protein substrates occurs through a mechanism similar to MrpL32.

Another example of limited proteolysis includes Atg32 protein, the mitochondrial OM-localized receptor involved in tagging mitochondria for autophagosomal degradation in yeast (Okamoto et al., 2009). The C-terminal domain of the protein is exposed into the IMS, where it is cleaved by the yeast *i*-AAA protease, Yme1 (Table 2) (Wang et al., 2013). This process generates the mature form of Atg32 that directly interacts with Atg11 and Atg8 proteins, leading to the formation of mitophagosome (Okamoto et al., 2009). There is no close Atg32 homolog in *A. thaliana*, and no proteins on the plant mitochondrial outer membrane with similar function have been experimentally confirmed to date. Recently, Broda et al. (2018) used a bioinformatic tool to identify *in silico* the Arabidopsis mitochondrial proteins that are specifically recognized by the Atg8 protein, a central protein of the autophagy machinery, based on the presence of a short Atg8-interacting motif (AIM) (Xie et al., 2016). Among the AIM-containing proteins, there are outer membrane proteins, which may play the role of mitochondrial receptors in the activation of mitophagy (Broda et al., 2018). It can be assumed that similarly to yeast, the Arabidopsis Yme1 ortholog, FTSH4 protease, activates mitophagy through proteolytic cleavage of specific OM-located substrates, but this needs to be confirmed. So far, a few proteins have been identified as proteolytic substrates of FTSH4, but there is no experimental evidence about the processing activity of this enzyme in plants (Opalinska et al., 2017, 2018; Maziak et al., 2021).

OMA1 protease has been initially identified in yeast as the mitochondrial inner membrane protease capable of replacing *m*-AAA in the proteolytic degradation of Oxa1 protein (hence original name overlapping with the *m*-AAA protease 1) (Käser et al., 2003). The homologs of OMA1 have been found in both prokaryotes and eukaryotes, with some exceptions, such as Drosophilidae, Nematoda, and Trematoda (Levytskyy et al., 2017). OMA1 is a metalloprotease containing a conserved Zn^2+^-binding motif (HExxH), but unlike the *i*-AAA and *m*-AAA proteases, it lacks the AAA domain and thus is ATP-independent. In yeast and mammals, the enzyme is mainly dormant under normal conditions; however, heat and oxidative stress and the loss of mitochondrial inner membrane potential lead to rapid activation of OMA1 (Baker et al., 2014; Bohovych et al., 2014; Zhang et al., 2014). It has been reported that the yeast Oma1 mediates degradation of newly synthesized, non-assembled Cox1 in cells lacking the Coa2 assembly factor (Khalimonchuk et al., 2012). The functional complementation assay demonstrated that the Arabidopsis OMA1 could partially replace the degradation function of the yeast protease (Migdal et al., 2017). There is no knowledge about the Oma1 processing activity in yeast to date. In addition to its significant role in the maintenance of mitochondrial bioenergetics, ultrastructure, and the stability of respiratory complexes in yeast, mammals, and plants (Bohovych et al., 2015; Migdal et al., 2017; Viana et al., 2021), OMA1 has been well recognized as a significant player, which together with the *i*-AAA protease controls the processing of mitochondrial dynamics regulator OPA1 in mammals (Table 2) (Griparic et al., 2007; Ehses et al., 2009; Head et al., 2009; Anand et al., 2014). As stated earlier, OPA1, and its yeast homolog Mgm1, are small GTPases that mediate mitochondrial fusion. In yeast, however, Mgm1 is proteolytically cleaved by rhomboid protease Pcp1 (van der Bliek and Koehler, 2003).

Generally, in mammals, OPA1 is present in different isoforms known as long OPA1 (L-OPA1), which represents an unprocessed form, and short OPA1 (S-OPA1) obtained by the proteolytic cleavage at two different cleavage sites, S1 and S2, by OMA1 and YME1L proteases, respectively (Anand et al., 2014). It has been displayed that the cleavage at the S1 is performed at the position R194-A195, while at the S2 - within the 217LQQQIQE223 sequence (Ishihara et al., 2006). Under non-stress conditions, YME1L mediates the processing of some of the L-OPA1 isoforms giving relatively equal amounts of L-OPA1 and S-OPA1, which is required to maintain the balance between mitochondrial fusion and fission. Under metabolic changes or mitochondrial dysfunction, the fission of mitochondrial inner membrane is triggered by activation of OMA1, which mediates rapid cleavage of all L-OPA1 isoforms into the short variants leading to the subsequent fragmentation of the mitochondrial network. This process enables the segregation and removal of damaged mitochondria through mitophagy (Anand et al., 2014). On the other hand, impaired activity of OMA1 prevents mitophagy by stabilizing L-OPA1, thus hindering mitochondrial fragmentation (MacVicar and Lane, 2014). Furthermore, OMA1 may also regulate apoptosis by mediating OPA1 proteolysis, leading to mitochondrial fission and leakage of proapoptotic factors in ATP-depleted renal tubular cells (Xiao et al., 2014).

Recently, Guo et al. (2020) have identified OMA1 as one of the molecular components of the integrated stress response (ISR) pathway in mammalian cells that signals mitochondrial stress to the cytosol. The ISR involves OMA1-dependent cleavage of the mitochondrial inner membrane protein DELE1 to its shorter form (DELE1_s_), which accumulates in the cytosol and triggers the stress response. The cleavage of DELE1 occurs at histidine 142 and does not require a specific sequence motif (Guo et al., 2020).

Lately, mitochondrial small heat-shock proteins (sHSPs) have been found to be under proteolytic control of the Arabidopsis OMA1 and FTSH4 proteases when plants are grown under prolonged moderate heat stress (Maziak et al., 2021). Under these conditions, sHSPs, identified in insoluble mitochondrial protein aggregates, were subjected to complete degradation rather than limited proteolysis as shown by the *in vitro* degradation assay in the Arabidopsis mitochondria in the presence of externally added OMA1 or FTSH4 synthesized in the cell-free expression system. The OMA1- and FTSH4-dependent proteolytic control of sHSPs appears to be unique to plants since organelle-targeted small heat-shock proteins have been so far identified in plants only, with an exception for a mitochondrion-targeted sHSP22 in *Drosophila melanogaster* (Maziak et al., 2021). There is no knowledge about the OMA1 processing activity to date in plants.

## Methods for Characterization of Mitochondrial Protein N-Termini, Protease Recognition Motif, and Proteolytic Substrates

Proteolytic processing produces two polypeptide chains where one possesses a protease-generated neo-N-terminus and the second a neo-C-terminus. Generated protein termini provide precise information about a protease cleavage site and specify a recognition motif within the protein substrates. The current proteomic methods to study native and neo termini of proteins (i.e., before and after proteolytic cleavage) are mass-spectrometry (MS) based N- and C-terminomics techniques, which facilitate identification of the N- and C-terminomes from the total proteomes. These techniques mostly rely on the bottom-up proteomics, in which a site-specific externally added protease digests proteomes to generate peptides before chromatographic separation and tandem MS (MS/MS) analysis, in contrast to the top-down proteomics approach, which directly analyzes intact proteins with all retained modifications by MS (Aebersold and Mann, 2016). Various proteomics technologies for N- and C-terminal analysis have been recently comprehensively described (reviewed in Tanco et al., 2015; Demir et al., 2018; Kaleja et al., 2019; Luo et al., 2019; Perrar et al., 2019; Bogaert and Gevaert, 2020; Kaushal and Lee, 2021; Mintoo et al., 2021). Due to some technical difficulties (e.g., poor ionization efficiencies of protein C-termini, low yielding carboxyl group labeling strategies), C-terminomics technologies are, however, less successfully implemented and widely used, in comparison to the N-terminomics methods, which in recent years have become an indispensable tool in positional proteomics (Eckhard et al., 2016).

Historically, the study of N-terminal protein sequences has been performed using the Edman degradation method, also known as N-terminal protein sequencing (Edman et al., 1950). For example, this method has been applied to determine the N-termini of potato tuber and Arabidopsis mitochondrial proteins (Jänsch et al., 1996; Millar et al., 1998; Kruft et al., 2001). Because of its limitations in automatization and inability to sequence N-terminally labeled proteins, the Edman degradation method became impractical for the N-termini characterization and was replaced by high-throughput MS-based N-terminomics technologies.

Generally, in N-terminomics, the strategies for selective isolation of N-terminal peptides from other digested internal peptides rely on two critical objectives. First, they utilize the unique reactivity of the primary amine at the protein N terminus, making the amine labeling highly favorable, in contrast to the less reactive carboxyl group (Mintoo et al., 2021). Second, they depend on selective enrichment of labeled N-terminal peptides by employing either a positive or negative selection approach, which reduces the complexity of a peptide sample. A positive selection strategy relies on a direct enrichment of N-terminal peptides from the peptide mixture [e.g., the Subtiligase and Chemical Enrichment of Protease Substrates (CHOPS) methods]. In the negative selection methods, the N-terminal peptides are enriched indirectly by the removal of undesired (internal, C-terminal) peptides (e.g., COFRADIC, ChaFRADIC, and TAILS methods) (Bogaert and Gevaert, 2020).

**FIGURE 3:**
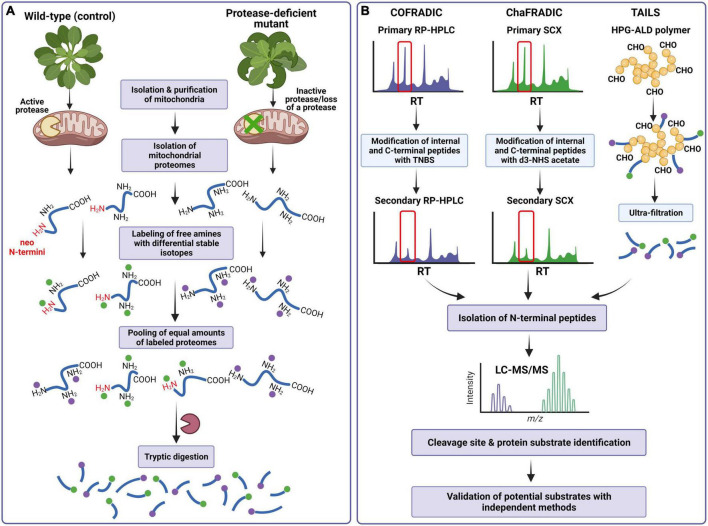
Scheme of the N-terminomics workflow for identifying a protease cleavage site and processing substrates using COFRADIC, ChaFRADIC, and TAILS as negative selection approaches for isolating N-terminal peptides in mitochondrial proteome studies. **(A)** Mitochondrial proteins isolated from the control and protease-deficient plants (containing protein neo-N- and native N-termini, respectively) are subjected to labeling all primary (α and ε) amines using stable differential isotopes to mass-tag native and neo-N-terminal peptides. Samples are pooled and digested by trypsin together. As a result, new primary α-amines at the N-termini of internal and C-terminal peptides are generated. **(B)** In COFRADIC and ChaFRADIC, the obtained peptides undergo the first separation by reversed-phase high-performance liquid chromatography (RP-HPLC) (COFRADIC) or by strong cation exchange (SCX) chromatography (ChaFRADIC). All collected peptides are modified with TNBS (in COFRADIC) or d3-NHS (in ChaFRADIC) to alter the retention time of internal and C-terminal peptides. The peptides are then subjected to the second identical chromatography step, in which only previously labeled N-terminal peptides are collected. In the case of TAILS, tryptic peptides are incubated with a polymer HPG-ALD. In this reaction, only internal peptides will bind to the polymer. The polymer-bound internal peptides are removed by centrifugation, while the N-terminal peptides are further recovered by filtration. In each type of experiment, the N-terminal peptides are analyzed by LC-MS/MS, which provides the protease cleavage site and identifies processing protein substrates. COFRADIC, combined fractional diagonal chromatography; ChaFRADIC, charge-based fractional diagonal chromatography; TAILS, terminal amine isotope labeling of substrates; TNBS, 2,4,6-trinitrobenzenesulfonic acid; d3-NHS, trideutero *N*-hydroxysuccinimide; HPG-ALD, high molecular weight polyglycerol aldehyde polymer; LC-MS/MS, liquid chromatography-tandem mass spectrometry. Details regarding specific methodologies are described in the main text. Based on Demir et al. (2018). Created with BioRender.com.

This chapter provides a short overview of a few N-terminomics techniques based on the negative selection approach, COFRADIC, ChaFRADIC, and TAILS, with their strengths and limitations. These methods have been successfully applied to generate global profiling of cellular and organellar N-terminomes, describe protease cleavage patterns, and identify proteolytic substrates in yeast, mammalian, and plant mitochondria (Figure 3).

### Combined Fractional Diagonal Chromatography

The N-terminal COFRADIC (combined fractional diagonal chromatography) is one of the earlier bottom-up proteomics technologies to enrich the N-terminal peptides. COFRADIC was initially developed by Gevaert et al. (2003), who introduced the concept of diagonal reversed-phase (RP) chromatography for isolating protein N-termini by depleting the internal peptides. The general principle of COFRADIC is as follows. After isolation and denaturation of studied proteomes, cysteine residues are first reduced and alkylated. This sample preparation step is commonly used in many, if not all, N-terminomics studies (Bogaert and Gevaert, 2020). Subsequently, all primary amines [α- and ε-amines of protein N-terminus and lysine (Lys) residues] are labeled using N-hydroxysuccinimide (NHS) acetate ester in *N*-acylation reaction. At this stage, it is possible to employ differential stable isotopes of NHS to mass-tag native and neo-N-terminal peptides allowing the distinction between different protein samples (e.g., protease-inactive mutant *versus* wild-type) (Staes et al., 2011) (Figure 3A). The differentially labeled samples are pooled and digested by trypsin together. Typically, trypsin cleaves peptide bonds at the C-terminal end of Arg and Lys residues; however, here, the digestion generates only Arg-ending peptides, as trypsin does not recognize acetylated lysine residues. The obtained peptides undergo the first separation by reversed-phase high-performance liquid chromatography (RP-HPLC), in which each peptide elutes after a particular retention time (RT) (Figure 3B). All collected peptides are then reacted with 2,4,6-trinitrobenzenesulfonic acid (TNBS). In this reaction, only internal and C-terminal peptides will be blocked because of the formation of a very hydrophobic 2,4,6-trinitrophenyl (TNP) group at their free α-amines. Each peptide fraction is then run on the second RP-HPLC using identical conditions as the primary run. However, since the strong hydrophobicity of the TNP group leads to a shift (an increase) in retention time of the TNBS-modified peptides, they elute later than in a primary run. In contrast, the peptides with labeled native and neo-N-termini have unaltered hydrophobicity and elute within the same time intervals as in the primary HPLC run. The native and neo-N-terminal peptides are collected and identified by liquid chromatography-tandem mass spectrometry (LC-MS/MS) (Figure 3B), providing the identity of protease substrates and protease cleavage sites (Staes et al., 2011; Luo et al., 2019; Mintoo et al., 2021).

So far, COFRADIC has been used in a broad spectrum of studies in different organisms. For example, this technique has been employed to identify physiological substrates of metacaspase 9 (MC9) in *A. thaliana* seedlings (Tsiatsiani et al., 2013) as well as for the identification, in combination with ribosome profiling, of novel translation initiation sites in Arabidopsis (Willems et al., 2017). This technique has also been employed to characterize protein substrates of HhoA, HhoB, and HtrA proteases in cyanobacterium *Synechocystis* sp. PCC 6803 (Tam et al., 2015). In humans, COFRADIC has been used to determine cleavage specificity and substrate identities of caspases (Van Damme et al., 2005; Wejda et al., 2012) and HIV-1 protease (Impens et al., 2012).

Notably, the COFRADIC method has been applied in the global analysis of the yeast *S. cerevisiae* mitochondrial N-terminome (Vögtle et al., 2009). In this landmark study, the N-termini of 615 mitochondrial proteins has been identified. Some mitochondrial proteins exhibited two N-termini differing by one amino acid residue. This observation led to identifying the Icp55 peptidase responsible for cleaving a single amino acid from −2R presequences following MPP cleavage, converting unstable intermediates into stable proteins. This study has also solved the controversial problem of the two MPP cleavage motifs (−2R and −3R), revealing that the −3R does not constitute an MPP cleavage site motif. Instead, it is a part of a motif for the two-step cleavage, first by MPP and then by Icp55 (Vögtle et al., 2009).

Furthermore, in this work, new protein substrates of another processing protease, Oct1, have also been identified (Vögtle et al., 2009). In a follow-up study, a comprehensive analysis of the N-termini of the Oct1 substrate intermediates compared to mature proteins revealed the presence of a destabilizing N-terminal amino acid in the intermediate form. This finding has uncovered that the processing by Oct1 converts unstable precursor intermediates generated by MPP into stable proteins (Vögtle et al., 2011).

To date, there is no information on the use of COFRADIC in identifying protein N-termini and processing substrates in plant mitochondria.

Since its original publication, some critical improvements to the COFRADIC protocol have been made. It has been observed that, after digestion, internal peptides containing the N-terminal glutamine residues may form spontaneously cyclic *N*-pyroglutamyl peptides, which do not react with TNBS and thus cannot be removed in the follow-up sorting process. To prevent it, two enzymatic reactions with glutamine cyclotransferase and pyroglutamyl aminopeptidase have been introduced, which eliminate pyroglutamate residues and expose a new α-primary amine for the TNBS reaction (Staes et al., 2008). Furthermore, an additional pre-enrichment step based on the strong cation exchange (SCX) chromatography at a pH of 3 for blocked (e.g., acetylated) N-terminal peptides has been introduced before the first RP-HPLC runs (Staes et al., 2008). Finally, an extra oxidation step of methionine with hydrogen peroxide (H_2_O_2_) has been included between the primary and secondary RP-HPLC separations and after TNBS modification. This step causes a shift in the retention time to an earlier point of the N-terminal methionine-containing peptides, segregating them from the non-methionine-containing N-terminal peptides (Gevaert et al., 2002; Van Damme et al., 2009). Despite being successfully employed in a wide range of applications, COFRADIC also has some limitations, which include the requirements for specific instruments and significant expertise as well as a relatively large amount of protein as a starting material (1–3 mg) (Perrar et al., 2019; Mintoo et al., 2021).

### Charge-Based Fractional Diagonal Chromatography

Charge-based fractional diagonal chromatography is a modified version of COFRADIC, which has been developed by the group of Venne et al. (2013) and for the first time implemented in the studies of the yeast mitochondrial N-terminome. This method utilizes the altered charged state of the tryptic peptides to separate native and neo-N-terminal peptides from the peptide mixture rather than their modified hydrophobicity. In addition, peptides are fractionated by SCX chromatography instead of RP-HPLC used in COFRADIC (Figure 3B). To label all primary amines, the isolated and then reduced proteins are dimethylated using formaldehyde. At this point, the incorporation of stable isotope dimethyl labeling (e.g., heavy and light dimethyl labels) enables quantitative differential analysis of two types of samples (Figure 3A). After pooling the samples and digesting them by trypsin, which hydrolyzes the peptide bonds C-terminal of Arg only, the peptide mixtures are separated by first SCX chromatography run at a pH of 2.7 (Figure 3B). After the first SCX run, which allows for selective separation of charged peptide fractions +1, +2, +3, +4, and >+4, all fractions are collected and treated with trideutero *N*-hydroxysuccinimide (d3-NHS) acetate (Venne et al., 2013; Bogaert and Gevaert, 2020). Only internal and C-terminal peptides with free α-amines are acetylated in this reaction, while the N-terminal peptides with blocked (i.e., labeled) α-amines remain unchanged. Since acetylation alters the charge of internal and C-terminal peptides, these modified peptides elute earlier in the second SCX chromatography run than during the first separation. The retention time of N-terminal peptides remains the same, allowing selective collection of native and neo-N-terminal peptides (Bogaert and Gevaert, 2020; Mintoo et al., 2021). The follow-up LC-MS/MS analysis provides the protease cleavage site and identifies processing protein substrates (Figure 3B).

Although ChaFRADIC, like COFRADIC, requires specific instruments and expertise, it is generally less labor-intensive. However, the main advantages, such as reductions in the amount of starting material (50–200 μg) and LC-MS/MS operation time, make ChaFRADIC more cost-effective than COFRADIC (Perrar et al., 2019; Mintoo et al., 2021). Additional improvements have been implemented in the past years, increasing this strategy’s power, sensitivity, and reproducibility. For example, Venne et al. (2015) have extended ChaFRADIC workflow by iTRAQ (Isobaric Tags for Relative and Absolute Quantitation) labeling and multi-enzyme protein digestion with trypsin, GluC, and subtilisin to analyze the N-terminal proteome of *A. thaliana* seedlings. This approach led to the identification of novel N-termini with increased overall proteome coverage. Recently, further reduction in the amount of a starting material (less than 5 μg of protein per condition) has been introduced in a tip-version of ChaFRADIC (ChaFRA*tip*) by a replacement of the SCX columns with the pipette tips containing SCX resins (Shema et al., 2018).

As stated earlier, for the first time, ChaFRADIC has been successfully applied in a study of the yeast mitochondrial protein N-termini (Venne et al., 2013). This pioneering work has enabled the differential quantitation of 1,459 non-redundant N-terminal peptides between two *S. cerevisiae* protein samples within a much shorter time and only 50 μg of mitochondrial proteins as a starting material. Furthermore, in this work, a quantitative comparative analysis of the yeast wild-type and *icp55*Δ mutant mitochondrial N-proteomes led to the identification of 14 novel substrates of Icp55, which were not identified in a previous study (Vögtle et al., 2009).

The ChaFRADIC has also been employed to identify MPP protein substrates in yeast *S. cerevisiae* mitochondria (Burkhart et al., 2015). Using wild-type and *mas1* mutant (temperature-sensitive mutant of the MPP subunit Mas1) mitochondria, 66 novel MPP substrates have been identified, confirming the −2R position as a crucial determinant for MPP recognition and processing (Burkhart et al., 2015).

Further, Carrie et al. (2015) employed ChaFRADIC in the quantitative comparative N-terminome analysis of *A. thaliana* mitochondria obtained from wild-type plants and mutants lacking ICP55 or OCT1 mitochondrial protease. The group has identified 88 mitochondrial proteins as putative ICP55 processing substrates and characterized the ICP55 protease cleavage motif, which appeared to be highly conserved between plants, yeast, and mammals. In this study, seven putative substrates of OCT1 protease were also identified. However, they did not display a consensus cleavage motif and did not reveal the presence of the classical −10R motif characteristic for other eukaryotes (Carrie et al., 2015).

### Terminal Amine Isotope Labeling of Substrates

Terminal Amine Isotope Labeling of Substrates was among the first N-terminomics methods developed. Initially introduced by the Overall lab (Kleifeld et al., 2010), TAILS has probably become the most widely used in N-terminome analyses. In short, the labeling of primary amines is typically performed through the dimethylation of N-terminal α- and ε-amines. At this step, stable isotope-labeled variants of formaldehyde can be introduced to label primary amines (Demir et al., 2017). Stable isotope labels can also be incorporated using amine-reactive isobaric reagents such as iTRAQ or tandem mass tags (TMT) (Figure 3A). Following trypsin digestion, the peptide mixture is incubated with a high molecular-weight dendritic polyglycerol aldehyde polymer (HPG-ALD) to separate N-terminal peptides from the internal peptides (Figure 3B). In this reaction, internal peptides covalently bind to the polymer, leaving the labeled N-terminal peptides unbound. The polymer-bound internal peptides are removed by centrifugation, while the N-terminal peptides are further recovered by filtration and identified by LC-MS/MS (Luo et al., 2019; Bogaert and Gevaert, 2020).

In the last years, TAILS has shown high robustness and reliability proven by the independent application of this method in numerous laboratories (Perrar et al., 2019). Furthermore, moderately low amount of a starting material (0.1–1 mg of protein per condition) and commercial availability of kits and reagents are additional advantages of TAILS. Despite these certain advantages, TAILS also has some limitations. For example, it requires expensive patented aldehyde polymer and significant statistical analyses to distinguish labeled and unlabeled N-termini in the complex peptide mixture (Mintoo et al., 2021). Following the principle of TAILS, the recent replacement of the HPG-ALD polymer by *N*-hydroxysuccinimide beads in a novel iNrich (integrated N-terminal peptide enrichment) workflow promises higher accuracy and suitability for deep N-terminome profiling (Ju et al., 2020).

The TAILS has been extensively used in N-terminome profiling and protease substrate identification in many different systems. In plants, TAILS has been employed to study the N-terminal acetylation of cytosolic and plastid-located proteins in the diatom *Thalassiosira pseudonana* (Huesgen et al., 2013) and the N-terminome of *A. thaliana* chloroplasts (Rowland et al., 2015). A similar approach has been taken for profiling the cleavage site of transit peptides for protein import into the cyanelles of the glaucophyte *Cyanophora paradoxa* (Köhler et al., 2015a) and chloroplasts of *A. thaliana* (Köhler et al., 2015b). Another TAILS-based study determined the impact of the Arg/N-end rule pathway on the Arabidopsis root proteome and the seed storage proteins (Zhang et al., 2015, 2018). So far, there is no information about the use of TAILS in the N-terminome analysis of plant mitochondria.

On the contrary, several TAILS-based studies have been applied to investigate the N-terminome of human mitochondria. For example, the modified version of TAILS termed MS-TAILS (mitochondrial SILAC-TAILS) has been used to analyze proteolysis in the mitochondria and parent cells during initial apoptotic events before caspase-3 activation (Marshall et al., 2018). This comprehensive approach has identified 206 mitochondrial proteins, which were not previously detected by shotgun analyses. It also has revealed 475 unique mitochondrial protein N-terminal peptides of all known mitochondrial proteins, with 97 previously unknown proteolytic sites, which constituted the highest reported coverage of the human mitochondrial N-terminome. Quantitative comparative analysis of mitochondrial and parental cell protein N-termini uncovered altered levels of mitochondrial proteins implicated in protein import, fission, and iron homeostasis, highlighting initial proteolytic events in the mitochondrial pathway during apoptosis (Marshall et al., 2018).

In another study, TAILS has been applied to profile the mitochondrial N-terminome and identify candidate mitochondrial protease activated by dopamine dysregulation in neuroblastoma cells during the early stages of Parkinson’s disease (PD) (Lualdi et al., 2019). The authors have identified eleven mitochondrial proteins with altered proteolytic processing, and one of these proteins, the 39S ribosomal protein L38, was cleaved by the neprilysin protease. In this study, for the first time, neprilysin has been identified as a protease linked with mitochondrial dysfunction upon the pathogenesis of Parkinson’s disease, suggesting exploring targets of neprilysin as candidate biomarkers of PD (Lualdi et al., 2019).

## Conclusion and Perspectives

The processing of mitochondrial proteins is a critical biological mechanism in maintaining organellar and cellular homeostasis by controlling protein location, abundance, and activity. Mitochondria house diverse biochemical pathways controlled by limited proteolysis, and many proteases are involved in protein processing. While regulating constitutive mitochondrial processes such as presequence cleavage and protein maturation is highly conserved and relatively well understood, the knowledge on condition-specific limited proteolysis is still far from complete (Table 3). For example, the removal of the mitochondrial targeting signals by MPP and IMP upon protein import and additional protein processing by ICP55 and OCT1 is evolutionarily conserved throughout diverse eukaryotes. The specific role of *m*-AAA in the biogenesis of mitochondrial ribosomes also seems to be preserved across yeast, mammals, and plants.

**TABLE 3:** Mitochondrial processes regulated by limited proteolysis in yeast, mammalian, and plant mitochondria.

Protease	Mitochondrial functions		
	Yeast	Mammals	Plants
MPP	Protein import and maturation		
ICP55	Protein import and maturation		
OCT1	Protein import and maturation		
IMP	Protein import and maturation		Unknown
ATP23	OXPHOS functionality	Unknown	Unknown
Rhomboid	Mitochondrial fusion/fission, morphology	Mitophagy, apoptosis	Unknown
*m*-AAA	Mitoribosome biogenesis		
*i*-AAA	Mitophagy	Mitochondrial fusion/fission	Unknown
OMA1	Unknown	Mitochondrial fusion/fission, integrated stress response (IRS), apoptosis	Unknown

*Details concerning the mitochondrial processing proteases and their protein substrates are provided in Table 2.*

On the other hand, substrate processing by ATP23, rhomboid, *i*-AAA, and OMA1 has rather diverse functions in yeast, mammals, and plants, suggesting that molecular mechanisms of limited proteolysis regulated by these mitochondrial proteases evolved differently in distinct eukaryote lineages. In yeast, the processing activity of rhomboid protease is required in mitochondrial fusion, and the *i*-AAA-mediated limited proteolysis is crucial in mitophagy. On the contrary, in mammals, the processing activity of *i*-AAA has been linked to mitochondrial fusion and fission, whereas the rhomboid protease controls mitophagy and apoptosis. The regulation of mammalian mitochondrial fusion and fission has also been associated with the processing activity of OMA1. Recent discoveries of the involvement of OMA1 in other processes, such as apoptosis and the integrated stress response (IRS), add new aspects to the understanding of the role of OMA1 as a critical processing protease in a variety of cellular processes in mammals.

Our understanding of the mitochondrial limited proteolysis in plants remains elusive despite numerous studies within the last years that have expanded our knowledge on the role of MPP, OCT1, ICP55, and *m*-AAA proteases in protein import and maturation. To date, there is no experimental evidence on the function of other mitochondrial proteases in limited proteolysis.

The characterization of protein N-termini generated from the proteolytic cleavage is crucial in uncovering novel protein substrates and understanding the physiological role of a protease. However, despite recent progress in discovering new processing peptidases and identifying protein processing as an additional mode-of-action of general quality control proteases, the substrate repertoire of such proteases is still relatively poor. This work provided a brief overview of a few N-terminomics techniques, COFRADIC, ChaFRADIC, and TAILS, which dominated the N-terminomics field until recently and were successfully employed to identify substrates of mitochondrial processing proteases.

The field of N-terminomics has been rapidly expanding over the last few years, promising more sensitive, faster, and more accessible methods in the precise annotation of the protease cleavage sites. Furthermore, the recent development of targeted degradomics, which allows the accurate quantification of cleaved products and an integrative approach combining N- and C-terminomics techniques, will provide a more comprehensive description of limited proteolysis and the role of proteases in regulating mitochondrial and cellular functions.

## Author Contributions

MH-C conceived and designed this study, prepared the illustrations, and wrote the manuscript. AM assisted with searching for literature and prepared the tables. HJ corrected the final edition. All authors read and approved the final version of the manuscript.

## Conflict of Interest

The authors declare that the research was conducted in the absence of any commercial or financial relationships that could be construed as a potential conflict of interest.

## Publisher’s Note

All claims expressed in this article are solely those of the authors and do not necessarily represent those of their affiliated organizations, or those of the publisher, the editors and the reviewers. Any product that may be evaluated in this article, or claim that may be made by its manufacturer, is not guaranteed or endorsed by the publisher.
